# TAM-ing T cells in the tumor microenvironment: implications for TAM receptor targeting

**DOI:** 10.1007/s00262-019-02421-w

**Published:** 2019-10-29

**Authors:** Marlies J. W. Peeters, Anne Rahbech, Per thor Straten

**Affiliations:** 1 National Center for Cancer Immune Therapy, Department of Oncology, University Hospital Herlev, Borgmester Ib Juuls Vej 25C, Copenhagen, Denmark; 2 Inflammation and Cancer Group, Department of Immunology and Microbiology, University of Copenhagen, Copenhagen, Denmark

**Keywords:** TAM receptors, MERTK, PROS1, T lymphocytes, Costimulation, CITIM 2019

## Abstract

The TAM receptors—TYRO3, AXL, MERTK—are pleiotropically expressed receptors in both healthy and diseased tissue. A complex of the ligands Protein S (PROS1) or Growth Arrest-Specific 6 (GAS6) with apoptotic phosphatidylserine activates the TAM receptors. Hence, this receptor family is essential for the efferocytosis of apoptotic material by antigen-presenting cells. In addition, TAM receptors are expressed by virtually all cells of the tumor microenvironment. They are also potent oncogenes, frequently overexpressed in cancer and involved in survival and therapy resistance. Due to their pro-oncogenic and immune-inhibitory traits, TAM receptors have emerged as promising targets for cancer therapy. Recently, TAM receptors have been described to function as costimulatory molecules on human T cells. TAM receptors’ ambivalent functions on many different cell types therefore make therapeutic targeting not straight-forward. In this review we summarize our current knowledge of the function of TAM receptors in the tumor microenvironment. We place particular focus on TAM receptors and the recently unraveled role of MERTK in activated T cells and potential consequences for anti-tumor immunity.

## Introduction

After the discovery of the TAM receptors (TYRO3, AXL, MERTK) three decades ago [1], many new functions of this receptor family have been discovered. Their expression is scattered across tissues, both in healthy and diseased, and their functions seem to be as ambivalent. TAM receptors are reported to be functionally expressed in sperm, neurons, platelets and retinal cells [2–5]. In addition, they are frequently overexpressed in cancer, and essentially abundant in innate and adaptive immune cells. Regardless of localization, TAM receptors are activated by their ligands Protein S (PROS1) and Growth Arrest Specific 6 (GAS6) which act as bridging molecules by binding to phosphatidylserine (PtdSer). Subsequently, the PtdSer-GAS6 or PtdSer-PROS1 complex fully activates the receptor. While GAS6 preferentially activates AXL, PROS1 has the highest affinity for MERTK and TYRO3 [6]. When activated, TAM receptor signaling can result in inhibitory or stimulatory responses depending on the cell context.

TAM receptors’ ambivalence becomes especially clear in tumor immunology. Their widespread expression in both cancer cells and various tumor-infiltrating immune cells can make conclusions indecisive. In this review, we will discuss the function of TYRO3, AXL and MERTK on all cells in the tumor micro-environment, with a special focus on T cells. The recently uncovered function of MERTK on activated T cells could have an important impact on anti-tumor immunity.

## TAM receptors in the tumor microenvironment

### Cancer

TAM receptors are reported to be overexpressed in both solid and hematological malignancies, and high expression of the TAM receptors has been associated with poor patient survival in a variety of cancers [7, 8]. Oncogenic TAM receptor signaling results in increased proliferation, cell survival and metastasis (reviewed in [7]). Many tumors express not only the receptors, but also the ligands PROS1 and GAS6 [9, 10]. In combination with the abundant presence of apoptotic PtdSer [11], cancer cells are capable of TAM receptor auto-signaling. This subsequently results in the activation of various downstream pathways, including ERK, MAPK, AKT and others [12, 13]. Interestingly, TAM receptors have been implicated in therapy resistance to MAPK, PI3 K and EGFR inhibitors [13]. Mechanistically, a study from Kotenko et al. showed that TAM receptor expression leads to upregulation of PD-L1, possibly contributing to therapy resistance [14].

As a result, TAM receptors seem an attractive target for cancer therapy, not in the least to overcome therapy resistance. Many small molecule inhibitors have been developed, which are usually reactive to all three TAM receptors [15]. Currently, AXL inhibitors are in clinical testing for NSCLC and pan-TAM inhibitors are in clinical testing for the treatment of metastasized solid tumors [8, 16]. In addition, many inhibitors are currently in the preclinical phase where they show anti-tumor activity (reviewed in [8]). Finally, MERTK is frequently overexpressed in leukemias, including T-ALL [15, 17]. Mouse studies show promising results where MERTK inhibition favors tumor regression [18]. In conclusion, TAM receptor targeting is a promising strategy for cancer therapy. However, since many other cell types express TAM receptors, inhibition of this integral receptor should be considered in the wider context of tumor-associated immune responses. It will be interesting to see how TAM receptor inhibition for cancer treatment develops, and caution might be warranted due to adverse immunological side effects.

### Antigen-presenting cells

Efferocytosis is the process of apoptotic cell clearance by antigen-presenting cells (APCs), including macrophages and dendritic cells (DCs). This is an essential process for tissue homeostasis. Due to regular cellular turnover, billions of apoptotic cells are removed by efferocytosis every day in the human body [19]. All three TAM receptors have essential functions in efferocytosis in APCs, which has been reviewed in detail by Rothlin and colleagues [20]. In short, TAM receptors in APCs are essential for efferocytosis and subsequent immune-suppressive signals. TAM receptor knockout mice have significant defects in the efferocytosis of apoptotic cells by APCs [21]. As a result, these mice show hyperactivation of APCs and subsequent autoimmunity.

TYRO3, AXL, and MERTK have distinct roles on APCs. Seitz et al. reported MERTK to be the main receptor for efferocytosis on macrophages, whereas AXL and TYRO3 were most essential for DCs [22]. In addition, Zagorska et al. showed MERTK to be a tolerogenic receptor in macrophages and during immunosuppression [23]. Correspondingly, they reported AXL to be an inflammatory response receptor on DCs, induced by proinflammatory stimuli. The molecular basis of TAM receptor signalling in APCs has been elucidated and functions mainly via downstream SOCS ubiquitin ligases and inhibition of Toll-like receptor signalling (reviewed in detail by Rothlin and colleagues [20]).

### NK and NKT cells

NK cells are important components of the innate immune system, as they provide rapid responses to both pathogens and tumors. TAM receptors play an important role in the differentiation of these cells. Caraux et al. showed that all three TAM receptors were expressed by maturing NK cells in mice [24]. This TAM receptor expression by NK cells was deemed essential for mouse NK cell maturation and subsequent expression of both inhibitory and activating receptors. It was also reported that AXL was essential for human NK cell development from hematopoietic progenitor cells [25]. The TAM receptors reportedly regulate NK cell development via IL-15, c-kit and T-BET [24, 25]. Furthermore, Paolino et al. reported that ubiquitination of the TAM receptors on mouse NK cells inhibited their proliferation and cytotoxicity in a tumor metastasis model [26].

Finally, MERTK has been reported to be functionally expressed by mouse NKT cells [27]. Genetic deletion of MERTK resulted in impaired cytokine secretion after antigen-induced activation. This was due to an intrinsic NKT cell defect, as the source of antigen-presenting cells (wild-type or MERTK-deficient) did not alter the impaired MERTK-deficient NKT cell response [27]. Taken together, all studies point toward an essential role for the TAM receptors in the functional maturation and cytotoxicity of NK(T) cells.

### B cells

In 2008, Shao et al. reported MERTK to be functionally expressed by wildtype mouse B cells. In their study, B cells upregulated MERTK upon chronic GvHD, while MERTK-deficient mice were protected against GvHD [28]. A few years later, the same group showed that upon MERTK depletion in mouse B cells, B cells decreased their antibody production and subsequent T cell activation [29]. Consistent with a stimulatory role for TAM receptors in B cells, both AXL and TYRO3 are reported to be overexpressed in B cell chronic lymphocytic leukemia patient samples [30, 31]. Correspondingly, AXL inhibition decreased B leukemia cell survival in vitro [30]. In conclusion, only a handful of researchers have studied the function of TAM receptors on B cells. The overall hypothesis links TAM receptor signaling to healthy B cell responses. Hence, dysregulation of TAM receptor signaling could lead to aberrant B cell proliferation and leukemia, although more research is needed.

### Platelets

TAM receptors on platelets have been well studied, and all three TAM receptors are expressed by both mouse and human platelets [2, 32, 33]. Various combinations of single, double, and triple TAM receptor knockouts have shown that all three TAM receptors are individually essential for normal platelet function [33]. Deletion of one of the TAM receptors resulted in mild dysfunctional platelets. Deletion of any two of the three TAM receptors did not affect platelet counts, but impaired hemostasis and platelet function. Lastly, absence of all three TAM receptors resulted in thrombocytopenia, severely impaired hemostasis and platelet function, and defective megakaryocytopoiesis [33]. In this regard, small-molecule MERTK-inhibition decreased mouse platelet activation and subsequent thrombosis [34]. Taken together, TAM receptor expression by platelets is essential for regular platelet function in both human and mouse.

## TAM receptors in T cells

### TAM receptor and ligand expression by T cells

T cells were long believed to be negative for TAM receptors. For the ligands, however, results have consistently showed expression by activated T cells. In 1997, it was shown for the first time that PROS1 expression was induced in mouse lymphocytes (both CD4^+^ and CD8^+^) upon TCR activation [35]. IL-4 addition further upregulated PROS1 expression and subsequent secretion. Almost two decades later, PROS1 expression in TCR-activated CD4^+^ T cells was confirmed by Carrera Silva et al. in both mouse and human [36]. Subsequently, Chan et al. showed that PROS1 expression in mouse CD4^+^ T cells was limited to the TH2 subset, and that PROS1 expression was dependent on IL-4 [37]. Regarding CD8^+^ T cells, our group recently verified PROS1 expression by human CD8^+^ T cells, as they expressed PROS1 upon TCR stimulation from day 2 onwards [38]. A more detailed overview of PROS1 expression in lymphocytes is found in Table 1. In all studies, resting T cells remained negative. Taken together, these studies prove that the TAM receptor ligand PROS1 is expressed and secreted by TCR-activated T cells. Interestingly, PROS1 was surface-bound in both ours and Carrera Silva’s study. This indicates that PROS1 is bound to either PtdSer or the TAM receptors, which could both be expressed by T cells. As for the other TAM receptor ligand GAS6, no expression by T cells has been reported.

**Table 1:** Evidence for TAM receptor and ligand expression by T lymphocytes

Cell type	Stimulant	TAM receptor/ligand expression	Additional findings	Species	Reference
CD3^+^ T cells from lymph node	αCD3 ± IL-4, 24 h	PROS1^+^	Resting CD4^+^ and CD8^+^ T cells were negative. IL-4 increased TCR-induced PROS1 expression.	Murine	[35]
CD8^+^ T cells from peritoneum	In vivo antigen activation by thymoma cancer cells	PROS1^+^	–	Murine	[35]
CD3^+^ T cells from PBMCs	PHA/PMA, up to 48 h	MERTK-	Directly ex vivo thymocytes negative for MERTK	Human	[17]
T cells from splenocytes	αCD3, up to 48 h	MERTK-	–	Murine	[27]
OT-II CD4^+^ T cells	OVA-DCs, 24 h	PROS1^+^	–	Murine	[36]
Naïve CD4^+^ T cells from PBMCs	αCD3/CD28, up to 6 days	PROS1^+^	Positive from day 3 onwards. Conserved in reactive lymph nodes (human)	Human	[36]
Naïve CD4^+^ T cells from PBMCs	αCD3/CD28, up to 7 days	MERTK^+^	Positive from day 3 onwards	Human	[40]
CD4^+^CD25^+^ Tregs from splenocytes	Directly ex vivo, no in vitro stimulation	AXL^+^ MERTK^+^	CD4^+^CD25− cells were negative	Murine	[41]
TH2 from splenocytes	αCD3/CD28 and TH2 polarization, 5 days	PROS1^+^	IL-4 dependent. TH1 and TH17 were negative	Murine	[37]
CD45^+^ TILs	Directly ex vivo, no in vitro stimulation	MERTK^+^	Gene expression from CD45^+^ TILs in the tumor micro-environment	Murine	[42]
CD4^+^ CD8^+^ T cells from PBMCs, healthy donors and SLE patients	Directly ex vivo, no in vitro stimulation	MERTK−/^+^, TYRO3−/^+^	Ex vivo, unstimulated T cells from healthy donors were negative for MERTK and TYRO3. CD4^+^ T cells from SLE patients had increased MERTK and TYRO3 expression. CD8^+^ remained negative.	Human	[39]
CD8^+^ T cells from PBMCs, CD3^+^ T cells from PBMCs	αCD3/CD28 up to 4 days, CMV/EBV/flu peptide stimulation for 48 h	PROS1^+^, MERTK^+^, TYRO3−/^+^ AXL−	Positive from day 2 onwards	Human	[38]

*SLE* systemic lupus erythematosus, *EVB* Epstein–Barr virus

In the early 2000s, two studies reported that T cells did not express the TAM receptors. Both studies reported no MERTK expression after two-day activation of mouse splenocytes with αCD3, or two-day activation of human T cells with PHA/PMA [17, 27]. In 2014, a study which reported increased MERTK and TYRO3 expression on CD4^+^ T cells from SLE patients went rather unnoticed [39]. The following year, Cabezon et al. convincingly showed that TCR-activated human CD4^+^ T cells expressed MERTK from day 3 onwards [40]. In addition, it was reported that murine CD4^+^ regulatory T cells expressed both AXL and MERTK, without in vitro or in vivo stimulation [41]. Regarding CD3^+^ T cells, Yokoyama et al. suggested that (mouse) CD45^+^ TILs could be responsible for increased MERTK levels in the tumor-microenvironment [42]. Finally, our group recently verified TAM receptor expression on human CD3^+^ and CD8^+^ T cells. We demonstrated on three different levels (RNA, protein, surface expression) that MERTK was expressed on TCR-activated human CD8^+^ T cells and CD3^+^ T cells [38]. In addition, we did not detect AXL and only a low amount of TYRO3.

The discrepancy of all later reports with the two earliest studies could be explained by the chosen species, timepoint, or stimulation method (a definitive overview is found in Table 1). Based on these studies, whether mouse T cells do or do not express any TAM receptor is until now not definitively proven. In humans, TAM receptor expression is better studied, especially regarding MERTK. Both Cabezon and our study showed that MERTK expression is only induced by TCR-mediated (e.g. via CD3 or peptide) activation and only detectable after 2 or 3 days [38, 40]. This could explain why Graham et al. found human T cells negative, as these were stimulated with non-TCR-specific PHA/PMA and the experiment did not go beyond 48 h [17].

According to our knowledge, only four studies have been published on MERTK expression on human T cells in the past 25 years (Table 1). The three most recent studies consistently found a varying amount and subset of T cells MERTK-positive. Combined with the independent and varying investigation methods used, these are compelling arguments for MERTK expression on primary T cells. Taken together, we conclude that TCR-activation leads to MERTK expression on both CD4^+^ and CD8^+^ human T cells. Combined with the T cell’s expression of PROS1, it becomes necessary to elucidate in what functional capacity the TAM receptors and ligands are expressed by T cells.

### TAM receptor function in T cells

Shortly after PROS1 was described to be expressed by mouse T cells, PROS1’s function on T cells was studied by the same group. Their study initially suggested that receptors for PROS1 transduced proliferative signals [43]. As the function and expression pattern of the TAM receptors was at that moment unknown, they attributed any positive or negative role to the anti-coagulant functions of PROS1 [43]. Their initial suggestion, however, that an Fc-TAM receptor competed with T cells for the ligand PROS1, proved to be correct two decades later. In this later study, Cabezon et al. added Fc-MERTK to CD4^+^ T cells. Subsequent PROS1 ligand depletion resulted in inhibition of T cell proliferation and activation [40]. Accordingly, adding exogenous PROS1 increased cytokine secretion and proliferation. This corresponds with our data on CD8^+^ T cells, where PROS1 positively regulated proliferation and cytokine secretion. We validated PROS1 signal transduction through MERTK using MERTK-inhibitors and knockdown of MERTK on CD8^+^ T cells [38]. As for GAS6, it has been reported that exogenous GAS6 could increase the suppressive properties of mouse CD4^+^ regulatory T cells via T cell-expressed AXL [41]. Furthermore, Keating et al. overexpressed MERTK in mouse T lymphocytes [44]. Their results showed that dysregulation of MERTK on T cells caused T cell leukemia due to uncontrolled division and proliferation. This highlights MERTK as a stimulatory T-cell molecule which, when dysregulated, results in disproportionate stimulatory and proliferative signals.

Since T cells have been believed not to express MERTK, previous results might need to be re-interpreted. To this end, it was previously shown that treatment of wildtype immunocompetent mice with MERTK-inhibitors decreased PD-1 expression on T cells [45]. PD-1 is solely expressed by previously activated T cells, is dependent on TCR signal strength and avidity, and is an identifying marker for tumor-reactive T cells [46]. T cells lacking PD-1 upon MERTK-inhibition are therefore likely to be less activated, non-tumor-reactive and correspondingly, less likely to respond to PD-1 immunotherapy. In agreement, we have shown with a MERTK-specific inhibitor that MERTK inhibition decreased T cell proliferation and cytokine secretion [38].

**Fig. 1:**
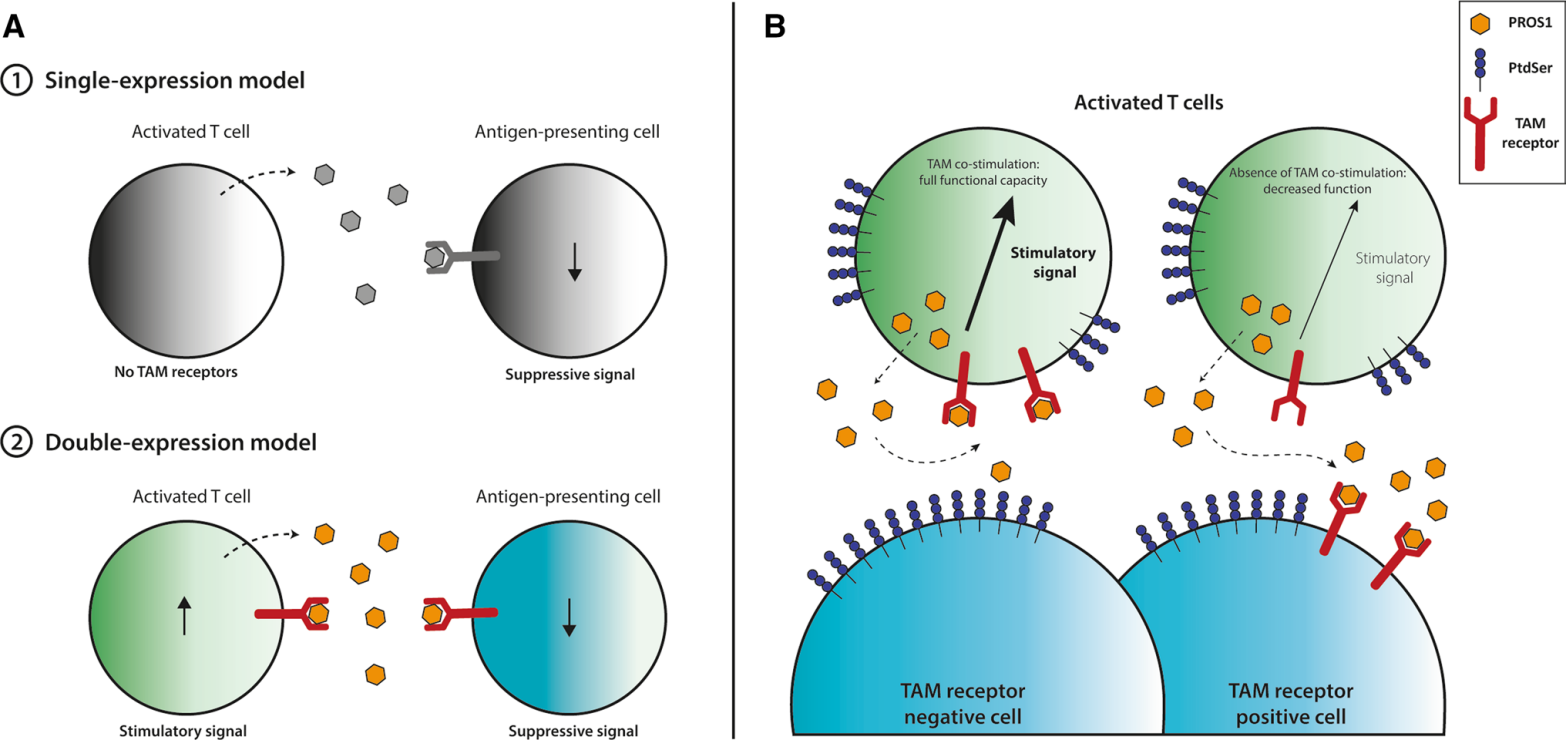
Consequences of TAM receptor signaling on T cells and competing cells. **a** Previously, T cells were thought to express PROS1, but not TAM receptors. PROS1 secretion by T cells subsequently resulted in a negative feedback loop to the APC. In the double-expression model, T-cell secreted PROS1 serves as an auto-stimulatory signal for the T cell and an inhibitory signal for the APC. **b** Activated T cells express PROS1, TAM receptors, and PtdSer. When the T cell encounters a TAM receptor negative cell, PROS1 will activate MERTK on the T cell. When the T cell encounters another TAM receptor positive cell, PROS1 will be competed for, as APCs and cancer cells express high amounts of TAM receptors. T cell-produced PROS1 will subsequently activate the other TAM receptor-positive cell (APC, cancer cell) and T cells will lose the PROS1-MERTK costimulatory signal. *PROS1* Protein S, *PtdSer* phosphatidylserine, *TAM receptor* TYRO3, AXL, MERTK receptor family

Finally, both Cabezon et al.’s and our study have indications that MERTK could regulate the T cell memory response. As MERTK seems to be a ‘late’ costimulatory molecule on T cells (only detectable from day 2–3 up to day 7—and possibly longer—after TCR activation), this is a tempting explanation. It will be exciting to see what future research uncovers. All in all, it becomes increasingly clear that the PROS1-MERTK axis has a functional impact on (human) T cells. Contrary to previous dogma, T cells do express TAM receptors and most importantly, MERTK seems a strong costimulatory receptor upon T cell activation (Fig. 1). This opens up exciting new areas in research for the TAM receptor field, as T cells are now added to the long list of TAM receptor-expressing cells. As availability of the ligands in vivo is not limitless, it will be important to pinpoint the net effect of TAM receptor signaling on the various immune subsets and cancer cells.

## TAM receptor targeting in the tumor microenvironment

As both cancer cells and immune cells express TAM receptors in various functional outputs, predicting the outcome of TAM receptor targeting in vivo is not straightforward. Hence, it is surprising that very few studies have been done regarding the immunological consequences of TAM receptor inhibition. Most of the studies have been performed in immunocompromised mice (reviewed in [8]). Only recently, the immunological consequences of TAM receptor inhibition for cancer treatment have gained more experimental attention [47]. These studies will become increasingly important as combination therapies with, e.g. immunotherapy are on the rise. With the many cell types expressing TAM receptors, the intended effect is not always trivial to predict. As a result, TAM receptor inhibitors for cancer therapy could result in unexpected effects in immunocompetent in vivo studies. For example, ablation of AXL and MERTK resulted in increased incidence of inflammatory colon cancer in mice [48]. In another model, TAM receptors on NK cells were found to positively regulate metastasis. Subsequent TAM receptor inhibition on NK cells, but not cancer cells, could decrease tumor burden [26]. Yet another study found that a pan-TAM receptor inhibitor for cancer treatment was only effective in immunocompetent mice, confirming the mode of action solely via effects on innate immune cells and not on the tumor [42].

Due to the TAM receptor inhibitors’ strong effect on APCs, TAM receptor inhibitors may well be interesting for their innate immunological effect, instead of their direct effect on cancer cells [47]. For AXL-specific or TYRO3-specific inhibitors, the desired anti-tumor immunological effect could be relatively easy to reach as these receptors dominantly function as immune-inhibitory receptors on APCs. AXL is implicated in therapy resistance and epithelial-mesenchymal transition, acts as a suppressive molecule on innate immune cells, and is absent as a costimulatory molecule on T cells [38, 47]. Therefore, targeting AXL using inhibitors is a very promising strategy through both direct effects on cancer cells, and indirect effects via activation of APCs and a subsequent anti-tumor immune response.

MERTK, however, has now emerged as a T-cell costimulatory molecule (Fig. 1). A previous study has shown that PROS1 ligand depletion by DCs will decrease T cell functionality [40]. This could be similar for the interaction of T cells with cancer cells. We have shown that low doses of PROS1 favor the tumor cells, by co-culturing MERTK-expressing T cells with high MERTK-expressing cancer cells [38]. Once saturating doses of PROS1 were reached—enough for both the T cells and the cancer cells—this effect was reversed as ligand competition was abrogated. In addition, MERTK-activation by exogenous PROS1 supplementation increased cytotoxicity of tumor infiltrating lymphocytes against autologous melanoma cells, despite high level of MERTK expression by the autologous tumor cell line.

Collectively, this leads us to believe that high TAM receptor-expressing cells (whether APCs or cancer cells) can compete with MERTK-expressing T cells for the PROS1 ligand in the tumor microenvironment or periphery (Fig. 1). MERTK inhibition subsequently decreases T cell functionality, whether through simple absence of the ligand or by direct inhibition. Collectively, this underscores the pitfalls of MERTK-inhibitors as cancer treatment, specifically in combination with T cell-based immunotherapy.

## Conclusion and perspectives

TAM receptors are potent oncogenes as underscored by numerous studies. Their overexpression plays an important role in therapy resistance, which prompted the development of TAM receptor inhibitors. While these show promising results in immunocompromised mice, studies in immune competent animals will be essential. As broad immunosuppressive signals in innate cells, TAM receptor inhibition could unleash an innate-driven anti-tumor immune response. Correspondingly, side effects could be severe. Triple TAM receptor knockout mice have massive lymphoproliferation, lupus-like symptoms, sterility, platelet dysfunction and inflammatory brain damage, among other symptoms [21, 49]. Blocking all three TAM receptors using inhibitors, should therefore be tightly controlled—if used at all—to prevent serious side effects. Loss or inhibition of only one of the TAM receptors seems more feasible.

As discussed earlier in this review, MERTK-specific inhibition could have ambiguous effects. TAM receptor inhibition has been suggested to be combined with T cell-based immunotherapy (e.g. anti-PD-1). Targeting MERTK would negatively affect T cell functionality, rendering PD-1 therapy counter-productive. It is tempting to speculate that activating MERTK on T cells would result in better T-cell stimulation and thus therapeutic outcomes. Direct administration of MERTK-agonists in vivo is not feasible, due to its overexpression in cancer. With the development of bi- or tri-specific antibodies and the improvements of CAR-, TCR-, or TIL-based T cell therapies, however, elucidating the mechanism of MERTK on T cells will be of great interest. Nevertheless, there are still many open questions. Until this date, it is unknown whether mouse T cells are truly negative for TAM receptor expression. Secondly, the mechanism of MERTK-mediated costimulation remains to be studied. Finally, definitive proof of MERTK’s involvement in generation of T cell memory and differentiation is yet to be unraveled. These questions open an exciting new era in TAM receptor research.

In conclusion, TAM receptors are attractive targets for cancer therapy, not in the least because of their suppressive actions in innate cells. Due to their widespread expression on many cells, including essential immune cells, the net results might not be as expected. We excitingly await future studies which determine if oncogenic TAM signaling in cancer cells, inhibitory TAM signaling in innate cells, or costimulatory TAM signaling in cytotoxic T cells, will tip the scale towards or against anti-tumor immunity.

## Abbreviations

GAS6: Growth arrest specific 6
MERTK: Mer tyrosine kinase
PROS1: Protein S
PtdSer: Phosphatidylserine
TAM receptors: TYRO3, AXL, MERTK receptors
